# Co-fermentation of the main sugar types from a beechwood organosolv hydrolysate by several strains of *Bacillus coagulans* results in effective lactic acid production

**DOI:** 10.1016/j.btre.2018.e00245

**Published:** 2018-03-05

**Authors:** Robert Glaser, Joachim Venus

**Affiliations:** Leibniz Institute for Agricultural Engineering and Bioeconomy, Max-Eyth-Allee 100, 14469 Potsdam, Germany

**Keywords:** MO, microorganism, Glc, glucose, Xyl, xylose, CB, cellobiose, LA, lactate, BM, biomass, DSMZ, Leibniz Institute’s German Collection of Microorganisms and Cell Cultures, Organosolv, Co-fermentation, Biorefinery, Growth model, Lactic acid

## Abstract

*Bacillus coagulans* is an interesting facultative anaerobic microorganism for biotechnological production of lactic acid that arouses interest. To determine the efficiency of biotechnological production of lactic acid from lignocellulosic feedstock hydrolysates, five *Bacillus coagulans* strains were grown in lignocellulose organosolv hydrolysate from ethanol/water-pulped beechwood. Parameter estimation based on a Monod-type model was used to derive the basic key parameters for a performance evaluation of the batch process. Three of the *Bacillus coagulans* strains, including DSM No. 2314, were able to produce lactate, primarily via uptake of glucose and xylose. Two other strains were identified as having the ability of utilizing cellobiose to a high degree, but they also had a lower affinity to xylose. The lactate yield concentration varied from 79.4 ± 2.1 g/L to 93.7 ± 1.4 g/L (85.4 ± 4.7 % of consumed carbohydrates) from the diluted organosolv hydrolysate.

## Highlights

• Batch cultivation of *Bacillus coagulans* using beechwood organosolv hydrolysate.
• Co-fermentation of glucose, xylose, and cellobiose in lactic acid production.
• Five strains of *B. coagulans* exhibited different abilities for cellobiose uptake.
• Growth performance evaluation using a Monod-type model.

## Nomenclature

*μ_BM,max_*: Maximum specific growth rate, mixed substrate
*μ_Glc,max_*: Maximum specific growth rate, glucose
μ_Xyl_ _max_: Maximum specific growth rate, xylose
*μ_CB,max_*: Maximum specific growth rate, cellobiose
*K_Glc_*: Monod saturation constant, glucose
*K_Xyl_*: Monod saturation constant, xylose
*K_CB_*: Monod saturation constant, cellobiose
*k_1_*: Kinetic constant
*k_2_*: Kinetic constant
*k_3_*: Kinetic constant
*Y^BM/ΔGlc^*: Biomass/glucose yield coefficient
*Y^BM/ΔXyl^*: Biomass/xylose yield coefficient
*Y^BM/ΔCB^*: Biomass/arabinose yield coefficient
*Y^BM/ΔALK^*: Biomass/alkaline yield coefficient
*Y^LA/ΔBM^*: Lactate/biomass yield coefficient
*Y^LA/Sub^*: Lactate/total substrate yield coefficient
*Y^LA/ΔSub^*: Lactate/consumed substrate yield coefficient
*C_Glc_*: Concentration of total initial glucose
*C_ΔGlc_*: Concentration of consumed glucose
*C_Xyl_*: Concentration of total initial xylose
*C_ΔXyl_*: Concentration of consumed xylose
*C_CB_*: Concentration of total initial cellobiose
*C_ΔCB_*: Concentration of consumed cellobiose
*C_Sub_*: Concentration of total initial substrate
*C_ΔSub_*: Concentration of consumed substrate
*dC_BM_/dt*: Biomass formation rate
*dC_Glc_/dt*: Substrate accumulation rate, glucose
*dC_Xyl_/dt*: Substrate accumulation rate, xylose
*dC_CB_/dt*: Substrate accumulation rate, cellobiose
*dC_LA_/dt*: Product formation rate, lactate
*q_0_*: Represents the physiological state of the inoculum
V_R_: Filled reactor volume

## 1 Introduction

Renewable biomass has been demonstrated to be suitable for the biotechnological production of biofuels and basic chemicals. Such biomass can be starchy biomass, sugar-based feedstocks, and lignocellulosic biomass [1]. The lignocellulosic feedstocks include agricultural residues, forest residues and wastes, fast-growing woods, such as hybrid poplar and willow, and herbaceous crops [[2], [3], [4]]. Lignocellulosic biomass requires a conversion process to make it suitable for further biotechnological processing [5,6]. Several pre-treatment methods exist to hydrolyse the bound carbohydrates – such as glucose, xylose, arabinose, galactose, and mannose – for fermentation. The pre-treatment methods that can be used to break down the structure of lignocellulosic biomass are categorized into five groups; i.e. physical treatment (e.g., mechanical disruption), chemical treatment (e.g., alkali, dilute acid, organosolv), thermal treatment (e.g., steam explosion), physicochemical treatment (e.g., ammonia fibre explosion, AFEX) and biological treatment (e.g., degradation by enzymes) [[7], [8], [9], [10]].

Most of the existing pre-treatment processes for lignocellulosic biomass lead to the formation of undesirable by-products that reduce the fermentation ability of hydrolysates, creating a major hindrance to product formation, such as furfural, 5‐hydroxymethylfurfural, and soluble lignin [[11], [12], [13], [14], [15], [16]].

Among the chemical degradation methods, the ethanol organosolv process is considered to be promising technology for the biorefining of lignocelluloses [17]. The organosolv process allows fractionation of lignocellulose fractions of cellulose, hemicellulose-derived monosugars, and the lignin [[18], [19], [20]]. Ethanol/water-based organosolv pre-treatment uses ethanol and water at elevated temperatures for a partial extraction of lignin and hemicellulose. The remaining cellulose fraction can be used for the production of monosugars by an enzymatic hydrolysis step, as the majority of lignin, the major hindrance to enzymatic hydrolysis, is removed [21]. Although the pre-treatment allows a relatively clear fractionation of the major wood components, the organosolv pre-treatment reaction conditions might cause the formation of complex compounds, such as furans and solvated phenolic components, residues of lignin, and organic acids. These residues are present in the different fractions of cellulose- and hemicellulose-derived sugars.

Methods to remove inhibitory compounds include the addition of activated charcoal, extraction with organic solvents, ion exchange or ion exclusion, molecular sieves, and treatment with laccases [12,14,[22], [23], [24], [25], [26], [27]]. Nonetheless, microorganisms (MO) that can endure inhibitory compounds in the fermentation medium are of industrial relevance. Here, the reduction of process costs can be achieved by lowering the necessity to achieve the lowest possible inhibitor concentration [28,29]. Such a reduction may be achievable using *Bacillus coagulans*.

*B. coagulans*, a spore-forming thermophilic facultative anaerobe and lactic acid producing bacterium, has the ability to grow at low pH [30] and is able to ferment hexose and pentose sugars to l‐(+)‐lactic acid with high titres [[31], [32], [33]]. Lactic acid (LA), long used in the food industry, has also become a common building block for chemical synthesis of the biodegradable polymer polylactic acid (PLA) [34]. With its versatile usability and the increasing demand for renewable bio-based plastics, there have been various attempts to produce LA efficiently in biorefineries using lignocellulosic feedstocks [35,36].

The scope of this study is to evaluate the performance of five *B. coagulans* strains on organosolv hydrolysate. The organosolv hydrolysate contains – besides potentially inhibitory compounds – glucose, xylose, and cellobiose as the predominant sugars that are fermented to lactate under thermophilic anaerobic process conditions. To derive the basic key performance parameters model-based parameter estimation is performed using a Monod-type model. The model give the opportunity to evaluate the bacterial growth according to key performance parameters such as maximum growth rates on the different carbohydrates and alkaline feed, as well as the impact of lactate on product inhibition of growth.

## 2 Materials and methods

### 2.1 Microorganisms

Five strains of *B. coagulans* were used for fermentation in organosolv hydrolysate, including *Bacillus coagulans* DSM No. 2314 and four other strains that were checked and identified as *B. coagulans* by the Leibniz Institute’s German Collection of Microorganisms and Cell Cultures (DSMZ). Those strains are further referred to and named as DSM ID 10-395, DSM ID 14‐298, DSM ID 14‐300, and DSM ID 14-301. Those strains are not purchasable through the DSMZ. The MOs were stored in cryogenic vials (VWR International GmbH, Germany) at −70 °C and reactivated on MRS broth (Merck KGaA, Germany) at 52 °C for 24 h. After full reactivation, the MOs were cultivated on slant culture tubes with MRS agar (Merck KGaA, Germany). Until use, the MOs were stored at 4 °C. The inoculum was cultivated on 60 mL MRS medium (Merck KGaA, Germany) in 250 mL shaking flasks (52 °C, 100 rpm, 15 h).

### 2.2 Growth medium

For growth medium, hydrolysate from the enzymatic hydrolysis of the cellulose fraction of organosolv pre-treated beechwood was used (charge number Kk002H1E1). The organosolv hydrolysate was provided by the Fraunhofer Centre for Chemical-Biotechnological Processes CBP (Leuna, Germany). Further information regarding the steadily improving organosolv process can be found in [18,20]. At the Fraunhofer Centre for Chemical Biotechnological Processes CBP, the lignocellulosic feedstock was pre-treated in a 460 L digester by an organosolv process using ethanol/water pulping at elevated temperatures at pilot scale. The solution used as a substrate for this work was produced after pre-treatment of beechwood chips at 170 °C using a 50 % (w.w.) ethanol/water solution containing 0.5 % sulphuric acid (based on dry wood). Enzymatic hydrolysis of the obtained pulp fraction was performed in a stirred tank reactor using 6 % of Cellic^®^ CTec2 and 0.25 % Cellic^®^ HTec2 (w./w. based on o.d. pulp) provided by Novozymes at 50 °C with a 10 % solids concentration for 48 h. The obtained sugar solution was concentrated using a falling film evaporator. The undissolved organosolv hydrolysate contained 307.5 g/L glucose, 72.1 g/L xylose, 85.3 g/L disaccharides, 9.6 mg/L hydroxymethylfurfural, 3.5 mg/L furfural and 7.7 g/L acetic acid (Section 2.5). The organosolv hydrolysate was used as a 1 to 4 dilution. For nutrition, the growth medium was supplemented with 15 g/L yeast extract.

### 2.3 Growth conditions

Cultivations were performed in a 2 L double-walled glass bioreactor Biostat B (Sartorius, Germany) with 1 L working volume. The growth medium solution was autoclaved at 121 °C for 20 min separately from the yeast extract to minimize the Maillard reaction. The pH was adjusted to 6.0 after autoclaving and controlled with a one-sided pH-control during the fermentation process using 20 % NaOH. The temperature was kept constant at 52 °C during fermentation. The inoculum was 2 % of the targeted working volume. Samples were taken at different time steps with a manual bypass system and inactivated for metabolite measurement in a hot water bath at 92 °C for 30 min [39]. After inactivation, the samples were stored at −8 °C for further use. After thawing the samples, they were centrifuged at 5000 rpm (relative centrifugal force (RCF) of 5338*g*) for 15 min at 4 °C. The supernatant was filtered with a 20 μm cellulose acetate membrane micro-filter (Th. Geyer GmbH & Co. KG, Germany). The filtrate was used for the detection of fermentable sugars using an HPLC (Section 2.5).

### 2.4 Biomass determination

The sample pellet was washed in 2.5 mL demineralized water, suspended, and centrifuged again at 5000 rpm (RCF = 5338*g*) and 4 °C for 15 min. While the supernatant was rejected, the pellet was placed in porcelain pots. The porcelain pots were previously dried for two hours at 105 °C. After cooling down in an exsiccator at a vacuum of 40 kPa for one hour, the tare weight of the porcelain pots was determined on a special accuracy-weighing machine. The pellet was suspended in demineralized water, transferred to the porcelain pots and dried under vacuum for at least 24 h at 105 °C. After cooling down onto room temperature, the pots were weighed. The procedure was repeated until the weight stayed constant. As a reference, see also DIN EN 12880. The averaged value of the two-fold weighing was used.

### 2.5 Detection of sugars, acids, and aldehydes

Sugar, lactate, and acetate concentrations were determined by High Performance Liquid Chromatography (HPLC) using a Dionex ICS 3000 (Thermo Fisher Scientific Inc., USA) equipped with an Eurokat H column (300 mm × 8 mm, 10 μm, eluent: 0.01 N H_2_SO_4_, including pre-column, Knauer GmbH, Germany) with an operation pressure of 65 bar. For detection, a refractive index detector RI-101 (SHODEX, Showa Denko Europe GmbH) was used. The column was operated at a constant temperature of 35 °C with a flow rate of 0.8 mL/min. The injection volume was 10 μL. For the previously mentioned fermentation conditions, lactic acid exists as a salt and therefore is reported as lactate [39]. Detection of aldehyde components was determined by HPLC using a Dionex ICS 3000 (Thermo Fisher Scientific Inc., USA) equipped with a Eurospher II C18 column (150 × 4 mm, pore size 100 Å, endcapped, including pre-column, Knauer GmbH, Germany) and a Dionex Series VWD UV/VIS-detector at 280 nm (Thermo Fisher Scientific Inc., USA). As eluent, ultrapure water (A) and 50 % acetonitrile solution (B) were used in a multistep gradient process. The multistep gradient elution was performed as follows: 7 min isocratic elution with 10 % B, 6 min gradient elution to 40 % B, 5 min gradient elution to 100 % B, 8 min isocratic elution with 100 % B, 4 min isocratic elution with 10 % B. The flow rate was set at 1 mL/min. The auto sampler temperature was 15 °C; the column and detector temperature were 23 °C. For evaluation of the chromatograms, the Chromeleon software version 6.80 (Thermo Fisher Scientific Inc., USA) was used.

## 3 Calculation

For the identification of the key performance parameters, a kinetic model of the additive Monod-type model for growth with mixed substrates, formerly described [[37], [38], [39]] was used to calculate the basic key performance indices such as growth rates, substrate consumption and product building rates. The model was adjusted to the need of this work. Here especially, the maintenance metabolism was not considered, as well as the nitrogen and phosphorous metabolism and oxygen conditions. Estimation of the model parameters was performed by minimization of the root mean squares (RMS) between the original experimental data (biomass-, glucose-, xylose-, cellobiose-, lactate concentrations, and alkaline amount used for pH-control) and model data using a generic method of MATLAB^®^ (The Mathworks, Natick, MA) optimization tools.

The data were further analysed through analysis of variance (ANOVA) using the null hypothesis, with the statement that all mean experimental values of the process and results of the model simulation are equal.

The dynamics in biomass concentration, *C_BM_*, were calculated as a function of the maximal growth rate, *μ_BM_*, and dependent on the dynamic change of the reactor volume, *V_R_*. Dilution occurs through alkaline feed, *F_Alk_*, for the control of the pH value.

(1) $$\frac{dC_{BM}\left(t\right)}{dt}=\mu_{BM}\cdot C_{BM}\left(t\right)-\frac{F_{Alk}\left(t\right)}{V_{R}\left(t\right)}C_{BM}\left(t\right)$$

The specific growth rate, *μ_BM_*, was described as a function of the diverse uptake rates of glucose, *μ_Glc_*, xylose, *μ_Xyl_*, and cellobiose, *μ_CB_*, while the theoretical maximum specific growth rate was defined as *μ_BM_*,*_max_*.

(2) $$\mu_{BM}=\mu_{BM,max}\cdot \left(k_{1}\mu_{Glc}+k_{2}\mu_{Xyl}+k_{3}\mu_{CB}\right)\cdot \left(\frac{1}{k_{1}+k_{2}+k_{3}}\right)$$

The coefficients *k_1_*, *k_2_*, and *k_3_* were described as theoretical parameters that are adjusted normally via parameter optimization. However, it was decided to set them to the corresponding uptake rates.

(3) $$\mu_{BM}=\mu_{BM,max}\cdot \left({\mu_{Glc}}^{2}+{\mu_{Xyl}}^{2}+{\mu_{CB}}^{2}\right)\cdot \left(\frac{1}{\mu_{Glc}+\mu_{Xyl}+\mu_{CB}}\right)$$

Due to product inhibition, the Monod-type model was extended with an inhibition term *I(LA)* to consider the effect of a critical lactate concentration, *C_LA,max_*, [40,41].

(4) $$I\left(LA\right)=\left(1-\frac{C_{LA}\left(t\right)}{C_{LA,max}}\right)$$

The product inhibition term was used as generalized nonlinear inhibition term *I(LA)^n^* using a power to the *n^th^* grade, referred to as “toxic power” [42], describing how the term of inhibition *(1 − C(t)/C_LA,max_)* strongly affects the specific growth and lactate production rates. A further term was used to take the differences of the duration of the initial lag phase into account until the MOs were ready for the uptake of the different sugar fractions. The term was described in [43] in the following form:

(5) $$\alpha_{i}\left(t\right)=(\frac{q_{0,i}}{q_{0,i}-exp\left(-\mu_{i,max}\cdot t\right)})\;\;\;\;with\;\;\;i=Glc,\;\;\;\;Xyl,\;\;\;\;CB$$

The dynamic equation to describe the glucose consumption, *C_Glc_*, was used in the following way:

(6) $$\frac{dC_{i}\left(t\right)}{dt}=-\frac{\mu_{i}}{Y^{BM/i}}\cdot C_{BM}\left(t\right)-\frac{F_{Alk}\left(t\right)}{V_{R}\left(t\right)}C_{i}\left(t\right)\;\;\;\;\;with\;\;\;i=Glc,\;\;\;Xyl,\;\;\;\;CB\;$$

with

(7) $$\mu_{i}=\mu_{i,max}\cdot \alpha_{i}\left(t\right)\cdot I{\left(LA\right)}^{n}\cdot \left(\frac{C_{i}\left(t\right)}{K_{i}+C_{i}\left(t\right)}\right)\;\;\;\;\;with\;\;\;\;i=\;Glc,Xyl,\;\;\;\;CB\;$$

The proposed product formation rate equation, *dC_LA_/dt*, was based on the simplified assumption that the rate of product formation was related to the rate of biomass formation through a production coefficient, *Y^LA/BM^*.

(8) $$\frac{dC_{LA}\left(t\right)}{dt}=Y^{LA/BM}\cdot \mu_{BM}\cdot C_{BM}\left(t\right)-\frac{F_{Alk}\left(t\right)}{V_{R}\left(t\right)}C_{LA}\left(t\right)$$

The value *Y^LA/BM^* can be derived through the relation *Y^LA/BM^* = (*Y^LA/Sub^/Y^BM/Sub^*). The reactor volume *V_R_* was set as dependent on the alkaline flow rate. The equation was derived from the dynamics of the pH-auxostat.

(9) $$\frac{dV_{R}\left(t\right)}{dt}=F_{Alk}\left(t\right)=\frac{\mu_{BM}\cdot V_{R}\left(t\right)\cdot C_{BM}\left(t\right)}{Y^{BM/Alk}\cdot C_{OH^{-}}}$$

For a further reduction of the models’ degrees of freedom, some parameters were dependent on each other: *K_Glc_* 1/*μ_Glc,max_*, *K_Xyl_* = 1/*μ_Xyl,max_*, and *K_CB_* = 1/*μ_CB,max_* as well as *K_D_* = *1/k_D_* [39]. The final model equation system was defined:

(10) $$\frac{dC_{BM}}{dt}=\mu_{BM,max}\cdot \left({\mu_{Glc}}^{2}+{\mu_{Xyl}}^{2}+{\mu_{CB}}^{2}\right)\cdot \left(\frac{1}{\mu_{Glc}+\mu_{Xyl}+\mu_{CB}}\right)\cdot C_{BM}\left(t\right)$$

(11) $$\frac{dC_{Glc}\left(t\right)}{dt}=-\frac{\mu_{Glc,max}\cdot {\left(1-\frac{C_{LA}\left(t\right)}{C_{LA,max}}\right)}^{n}\cdot \left(\frac{q_{0,Glc}}{q_{0,Glc}-exp\left(-\mu_{Glc,max}\cdot t\right)}\right)\cdot \left(\frac{C_{Glc}\left(t\right)}{K_{Glc}+C_{Glc}\left(t\right)}\right)}{Y^{BM/Glc}}\cdot C_{BM}\left(t\right)$$

(12) $$\frac{dC_{Xyl}\left(t\right)}{dt}=-\frac{\mu_{Xyl,max}\cdot {\left(1-\frac{C_{LA}\left(t\right)}{C_{LA,max}}\right)}^{n}\cdot \left(\frac{q_{0,Xyl}}{q_{0,Xyl}-exp\left(-\mu_{Xyl,max}\cdot t\right)}\right)\cdot \left(\frac{C_{Xyl}\left(t\right)}{K_{Xyl}+C_{Xyl}\left(t\right)}\right)}{Y^{BM/Xyl}}\cdot C_{BM}\left(t\right)$$

(13) $$\frac{dC_{CB}\left(t\right)}{dt}=-\frac{\mu_{CB,max}\cdot {\left(1-\frac{C_{LA}\left(t\right)}{C_{LA,max}}\right)}^{n}\cdot \left(\frac{q_{0,CB}}{q_{0,CB}-exp\left(-\mu_{CB,max}\cdot t\right)}\right)\cdot \left(\frac{C_{CB}\left(t\right)}{K_{CB}+C_{CB}\left(t\right)}\right)}{Y^{BM/CB}}\cdot C_{BM}\left(t\right)$$

(14) $$\frac{dC_{LA}\left(t\right)}{dt}=Y^{LA/BM}\cdot \mu_{BM}\cdot C_{BM}\left(t\right)-\frac{F_{Alk}\left(t\right)}{V_{R}\left(t\right)}C_{LA}\left(t\right)$$

(15) $$\frac{dV_{R}\left(t\right)}{dt}=F_{Alk}\left(t\right)=\frac{\mu_{BM}\cdot V_{R}\left(t\right)\cdot C_{BM}\left(t\right)}{Y^{BM/Alk}\cdot C_{OH^{-}}}$$

## 4 Results

Batch fermentation processes were performed on lignocellulose organosolv hydrolysate. The hydrolysate was obtained from the second organosolv fractionation procedure conducted in the pilot plant by the Fraunhofer Centre for Chemical Biotechnological Processes CBP in Leuna, Germany. The organosolv hydrolysate has an extremely high content of cellobiose that is not typical for this process and can be explained by a relatively incomplete enzymatic hydrolysis after the ethanol/water solvation. However, this cellobiose content is an interesting effect that allows investigation of the cellobiose fermentation ability (hydrolytic activity) of the *B. coagulans* strains used in this study.

To achieve comparable growth results between the strains, a supplement of 15 g/L yeast extract (compare to [45] using 20 g/L yeast extract at 240 g/L glucose) was used to exclude a nutritional lag, although the cost of such a medium would be too high for industrial purposes. A comparison study for the use of a cheap nutrition source using leguminous green juice was detailed [44].

**Fig. 1:**
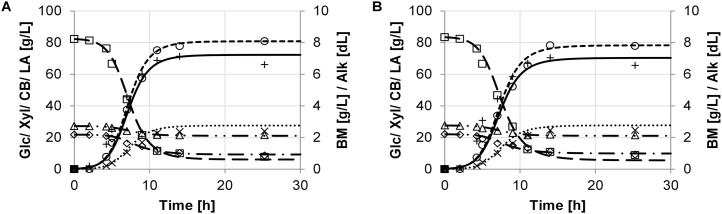
Fermentation of organosolv hydrolysate with strain DSM No. 2314. Experimental results are displayed as marks (□ glucose, ◊ xylose, (cellobiose, ○ lactate, + biomass, × (alkaline). Predicted simulation results are shown as lines (− − − glucose, − ▪ − ▪ xylose, — ▪ ▪ cellobiose, − − − lactate, − biomass, ▪ ▪ ▪ alkaline). Diagrams A and B refer to duplicate fermentations with the same inoculum.

**Fig. 2:**
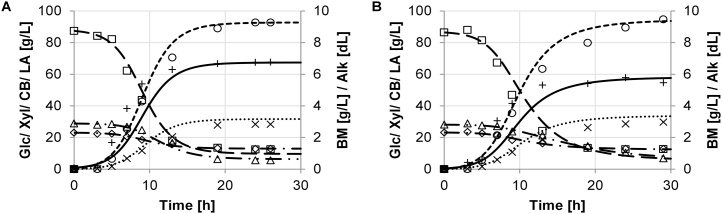
Fermentation dynamics of organosolv hydrolysate with strain DSM ID 14-301. Experimental results are displayed as marks (□ glucose, ◊ xylose, Δ cellobiose, ○ lactate, + biomass, × alkaline). Predicted simulation results are shown as lines (− − − glucose, − ▪ − ▪ xylose, − ▪ ▪ cellobiose, − − − lactate, − biomass, ▪ ▪ ▪ alkaline). Diagrams A and B refer to duplicate fermentations with the same inoculum.

**Fig. 3:**
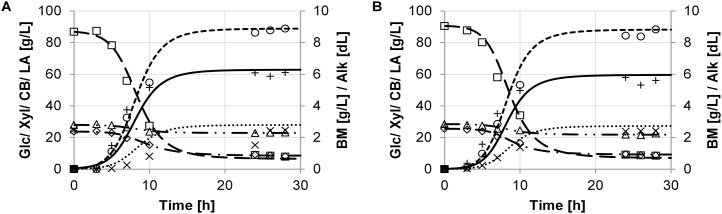
Fermentation dynamics of organosolv hydrolysate with strain DSM ID 14-300. Experimental results are displayed as marks (□ glucose, ◊ xylose, Δ cellobiose, ○ lactate, + biomass, × alkaline). Predicted simulation results are shown as lines (− − − glucose, − ▪ − ▪ xylose, − ▪ ▪ cellobiose, − − − lactate, − biomass, ▪ ▪ ▪ alkaline). Diagrams A and B refer to duplicate fermentations with the same inoculum.

**Fig. 4:**
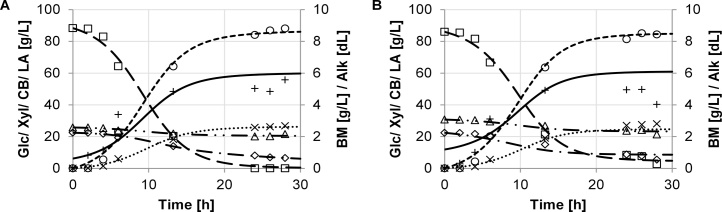
Fermentation dynamics of organosolv hydrolysate with strain DSM ID 14-298. Experimental results are displayed as marks (□ glucose, ◊ xylose, Δ cellobiose, ○ lactate, + biomass, × alkaline). Predicted simulation results are shown as lines (− − − glucose, − ▪ − ▪ xylose, − ▪ ▪ cellobiose, − − − lactate, − biomass, ▪ ▪ ▪ alkaline). Diagrams A and B refer to duplicate fermentations with the same inoculum.

**Fig. 5:**
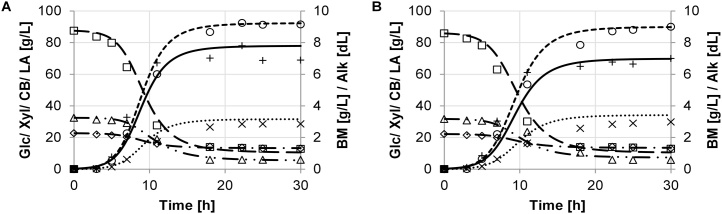
Fermentation dynamics of organosolv hydrolysate with strain DSM ID 10-395. Experimental results are displayed as marks (□ glucose, ◊ xylose, Δ cellobiose, ○ lactate, + biomass, × alkaline). Predicted simulation results are shown as lines (− − − glucose, − ▪ − ▪ xylose, − ▪ ▪ cellobiose, − − − lactate, − biomass, ▪ ▪ ▪ alkaline). Diagrams A and B refer to duplicate fermentations with the same inoculum.

The growth performance was evaluated by key performance parameters, which were determined based on the model described in section 3. The experimental data and the model are in good agreement. Batch growth of the different strains of *B. coagulans* and results of the model simulation are shown in Fig. 1, Fig. 2, Fig. 3, Fig. 4, Fig. 5. The experimental data are indicated as marks. Simulations are shown as dashed lines. The experimental derived and estimated model parameters are given in Table 1.

**Table 1:** Coefficients and parameters of growth derived from parameter fitting and experimental data that were used for the model simulation of bacterial growth shown in Fig. 1, Fig. 2, Fig. 3, Fig. 4, Fig. 5.

	DSM No. 2314		DSM ID 14-301		DSM ID 14-300		DSM ID 14-298		DSM ID 10-395		
Concentrations of biomass and sugar^a^											
C_Sub,max_	131.30	133.07	139.27	137.70	138.56	144.54	136.54	139.18	142.65	139.72	(g/L)
C_Sub,min_	41.52	43.81	30.76	35.79	39.09	39.63	28.17	29.48	31.84	33.17	(g/L)
C_∆Sub_	89.77	89.26	108.51	101.91	99.47	104.91	108.38	109.7	110.81	106.55	(g/L)
C_BM,min_	0.03	0.03	0.09	0.05	0.06	0.02	0.04	0.03	0.03	0.03	(g/L)
C_BM,max_	7.13	7.04	6.77	5.78	7.46	5.34	5.58	4.95	7.80	7.02	(g/L)
C_∆BM_	7.10	7.01	6.68	5.73	7.40	5.32	5.54	4.92	7.77	6.99	(g/L)
C_Glc,max_	82.25	83.42	87.41	86.43	86.83	90.55	88.33	86.00	87.49	85.81	(g/L)
C_Glc,min_	7.80	8.88	12.58	12.73	7.87	8.48	0.00	2.67	12.74	12.86	(g/L)
C_∆Glc_	74.45	74.54	74.83	73.70	78.96	82.07	88.33	83.33	74.75	72.95	(g/L)
C_Xyl,max_	21.81	22.05	23.04	23.15	23.75	25.53	22.45	22.44	22.70	22.18	(g/L)
C_Xyl,min_	8.66	9.11	12.90	12.82	8.07	8.69	6.55	5.45	12.98	13.10	(g/L)
C_∆Xyl_	13.15	12.94	10.14	10.33	15.89	16.84	15.9	16.99	9.72	9.08	(g/L)
C_CB,max_	27.23	27.59	28.83	28.12	27.97	28.46	25.72	30.74	32.45	31.72	(g/L)
C_CB,min_	21.43	21.40	5.59	6.80	23.14	22.47	21.61	21.36	5.85	5.77	(g/L)
C_∆CB_	5.80	6.19	23.24	21.32	4.83	5.99	4.11	9.38	26.60	25.95	(g/L)
C_LA,max_	80.87	77.90	92.73	94.69	88.88	88.27	88.09	84.34	91.41	89.96	(g/L)

Parameter derived from experimental data											
Y^BM/Sub^	0.0563	0.0551	0.0513	0.0444	0.0578	0.0396	0.0431	0.0389	0.0590	0.0533	(g/g)
Y^BM/Glc^	0.0508	0.0491	0.0435	0.0375	0.0523	0.0357	0.0431	0.0375	0.0498	0.0448	(g/g)
Y^BM/Xyl^	0.0333	0.0316	0.0209	0.0183	0.0372	0.0255	0.0297	0.0287	0.0233	0.0198	(g/g)
Y^BM/CB^	0.0108	0.0110	0.0408	0.0331	0.0077	0.0085	0.0069	0.0099	0.0477	0.0430	(g/g)
Y^BM/LA^	0.0890	0.0895	0.0719	0.0611	0.0858	0.0621	0.0629	0.0586	0.0834	0.0777	(g/g)
Y^LA/Sub^	0.6329	0.6162	0.7137	0.7282	0.6735	0.6381	0.6851	0.6622	0.7056	0.6859	(g/g)
Y^LA/*∆*Sub^	0.9008	0.8727	0.8546	0.9292	0.8935	0.8414	0.8128	0.7688	0.8249	0.8443	(g/g)
Y^BM/ALK^	8.0234	8.0653	6.4796	5.5023	7.7292	5.5956	5.6673	5.2762	7.3908	7.0015	(g/mol)

Parameter derived from parameter estimations											
μ_BM·max_	3.5016	3.7016	3.9806	4.0432	3.4428	2.9303	2.6813	3.1041	3.5141	3.6671	(1/h)
μ_Glc,max_	0.4860	0.4837	0.4315	0.4425	0.4815	0.4672	0.4676	0.4359	0.4463	0.4374	(1/h)
μ_Xyl,max_	0.4354	0.5223	0.1678	0.1360	0.3981	0.2888	0.2521	0.3399	0.1010	0.0699	(1/h)
μ_CB,max_	0.0346	0.0425	0.5755	0.5835	0.1000	0.0475	0.0941	0.0407	0.5093	0.5610	(1/h)
n	1.3101	1.4272	1.7037	2.0536	1.7174	1.8092	1.7513	1.7668	1.6691	1.8972	(-)
q_0/Glc_	6.3858	13.137	0.8519	0.8553	0.9914	5.5568	4.7900	4.5092	4.2238	7.1783	(-)
q_0/Xyl_	0.0064	0.0020	0.0936	0.1896	0.0062	0.0337	0.0405	0.0086	0.5596	2.5881	(-)
q_0/CB_	4.1315	2.3622	0.0008	0.0002	0.0335	0.9161	0.0403	1.1219	0.0034	0.0011	(-)

Estimation quality											
∆σ	1.8877	2.2562	2.0953	2.3217	2.3249	2.1171	2.8040	2.4756	1.6132	1.5015	
RMS	2.0626	2.0958	2.4347	2.6007	2.4518	2.0912	4.4515	3.2074	2.0948	2.4271	
R^2^	0.9991	0.9987	0.9975	0.9988	0.9987	0.9991	0.9976	0.9984	0.9994	0.9992	

ANOVA											
*F*	2.1E-5	2.6E-4	4.9E-4	8.0E-4	4.9E-4	2.8E-4	3.0E-4	8.8E-4	6.9E-4	7.3E-6	
*Fcritical*	3.9175	3.9175	3.9175	3.9175	3.9273	3.9273	3.9273	3.9273	3.9273	3.9175	
*p*	0.9964	0.9872	0.9990	0.9775	0.9824	0.9866	0.9861	0.9764	0.9791	0.9979	

a Data derived by HPLC measurement described in section 2.3 after inoculum addition.

For *B. coagulans* strain DSM No. 2314, a very short lag phase is evident (Fig. 1A and B). The exponential growth depends mostly on the consumption of glucose and xylose with an averaged difference in the concentration of 74.5 ± 0.1 g/L and 13.1 ± 0.2 g/L, respectively. Glucose and xylose was nearly metabolized in parallel. No intermediate lag phase could be observed. The cellobiose was not well-metabolized, with only a small decrease in concentration of 6.0 ± 0.3 g/L. A averaged yield of 79.4 ± 2.1 g/L (60.1 ± 1.9 %) LA was produced from 132.2 ± 1.3 g/L total substrate solution. This LA production equals 88.7 ± 2.4 % of a yield based on the total consumed sugar amount.

Fig. 1 Comparing the growth of DSM No. 2314 and DSM ID 14-301 (Fig. 2A and B), a slightly longer initial lag time and a lower maximum specific growth rate, *μ_BM,max_* = 4.6 ± 1.4 1/h, was observed for the second strain. DSM ID 14-301 showed good consumption of the xylose and even higher uptake of the cellobiose fraction. The strain was able to utilize 105.2 ± 4.7 g/L of the total available amount of carbohydrates of 138.5 ± 1.1 g/L. The result is a difference in concentration from the process start to the end of 74.3 ± 0.8 g/L glucose, 10.2 ± 0.1 g/L xylose, and 22.3 ± 1.4 g/L cellobiose. A yield concentration of 89.7 ± 1.4 g/L of lactate could be achieved. This equals a yield of 67.7 ± 1.3 % from the total available sugar input and 89.1 ± 4.6 % yield of the consumed sugar amount.

Although the DSM ID 14–301 strain had a slightly lower average biomass concentration (6.2 ± 0.7 g/L) than DSM No. 2314 (7.1 ± 0.1 g/L), only a slightly higher yield could be achieved. This is represented by the yield coefficient *Y^BM/ΔSubs^*, which was slightly higher than this yield coefficient of DSM No. 2314 (Table 1). That can be seen also using the average yield coefficient *Y^LAlΔSub^* (0.9 ± 0.1 g_LA_/g_ΔSub_) of DSM ID 14‐301, which was not significantly higher than for DSM No. 2314 (0.9 ± 0.1 g_LA_/g_ΔSub_). The molar yield coefficient of *Y^BMlAlk^* was lower in DSM ID 14‐301 at 6.0 ± 0.7 g_BM_/mol_Alk_ than in DSM No. 2314 (8.0 ± 0.3 g_BM_/mol_Alk_).

Fig. 2 Strain DSM ID 14–300 (Fig. 3A and B) gave similar results to DSM No. 2314. DSM ID 14–300 consumed glucose and xylose as the main carbohydrate sources, while cellobiose was only partially utilized. Despite the low biomass production (maximum of 6.4 ± 1.5 g/L), a comparable lactate yield of 88.6 ± 0.4 g/L could be achieved. The average yield for strain DSM ID 14–300 was found at 86.7 ± 3.8 % of the fermented sugars and 62.6 ± 3.0 % of total sugar amount.

Fig. 3 DSM ID 14–298 (Fig. 4A and B) showed a comparable biomass yield to DSM ID 14–300 with 5.2 ± 0.4 g/L. Nearly the entire amounts of glucose and xylose were consumed. However, this strain has a low affinity towards cellobiose as indicated by the small change in cellobiose concentration. DSM ID 14–298 had a mean yield of 67.4 ± 1.6 % of the total available sugar amount and 79.1 ± 3.1 % of the consumed sugars.

Fig. 4 Strain DSM ID 10–395 (Fig. 5A and B) performs well with cellobiose as a carbohydrate substrate but has a low affinity for xylose as shown by the small change in xylose concentration. While 73.9 ± 1.3 g/L glucose and 26.3 ± 0.5 g/L cellobiose were consumed, the xylose concentration only showed a decrease of 9.4 ± 0.5 g/L. A mean biomass concentration of 7.4 ± 0.6 g/L could be achieved. The yields were 64.2 ± 1.6 % of the total sugar and 83.4 ± 2.9 % of consumed sugar.

Fig. 5 The parameter fitting of the proposed model to the experimental data gave good results.

The overall mean standard derivation is *σ^2^* = 2.1 ± 0.4 while the mean RMS values are 2.6 ± 0.7. The overall averaged correlation coefficients are *R^2^* = 0.999 ± 0.001. The results of the ANOVA based on a 95 % confidence interval have an overall satisfying relation of *F < F_critical_*. With the *p*‐value of near *p*≅*1* with 0.987 ± 0.008, it could be assumed that the process data and model solution are significantly equal. Reported values of the model parameters differ depending on the used strain, but the values determined here were within the range of previously reported values [39].

The generalized nonlinear inhibition term considers that there is a determined concentration of product above which growth and production do not occur. In the model used here, the parameter *C_LA,max_* represents the lactate concentrations at which the growth and production processes are interrupted either due to the high lactate concentration itself or to a lack in nutrition. The nutrition with yeast extract lies normally in the range of 1–2 % of the carbohydrate source. The concentration *C_LA,max_* differs between the strains is it is expectable.

The growth model mirrors the characteristics of the strains. The maximum specific growth rates reveal that strain DSM ID 14‐301 has the fastest growth on biomass (*μ_BM,max_* = 4.01 ± 0.04 1/h) before strain DSM No. 2314 (*μ_BM,max_* = 3.60 ± 0.14 1/h) and strain DSM ID 10-395, with *μ_BM,max_* = 3.59 ± 0.11 1/h. Lower in specific maximum growth rates are strains DSM ID 14‐300 and DSM ID 14-298, with *μ_BM,max_* = 3.19 ± 0.36 1/h and *μ_BM,max_* = 2.89 ± 0.30 1/h (p = 0.05), respectively. Comparing the glucose uptake rates, they show a good comparability (Table 1) with a low overall standard deviation. The total average value of the glucose uptake rates (Table 1) is *μ_Glc,_*max = 0.46 ± 0.02 1/h. The deviation is barely 4.7 % between the strains, so the glucose uptake rates show a good comparability between the strains with a low overall standard deviation, demonstrating comparable performance on the substrate glucose. Comparing the xylose consumption, the strains with the higher consumed xylose amount also have higher uptake rates. Here, strain DSM No 2314 has the highest value, with *μ_Xyl,max_* = 0.48 ± 0.06 1/h. Despite a higher amount of consumed xylose, strains DSM ID 14‐300 and DSM ID 14‐298 had uptake rates with average values of *μ_Xyl,max_* = 0.34 ± 0.08 1/h and *μ_Xyl,max_* = 0.30 ± 0.06 1/h, respectively. For comparison, strain DSM ID 14‐301 and DSM ID 10‐395 had uptake rates in the range of *μ_Xyl,max_* = 0.152 ± 0.023 1/h and *μ_Xyl,max_* = 0.086 ± 0.022 1/h. This characteristic is comparable to the behaviour of the strains with better cellobiose consumption. Here, those cellobiose-using strains have a higher uptake rate than for glucose. The strains DSM ID 14‐301 and DSM ID 10‐395 had uptake rates in the range of *μ_CB,max_* = 0.58 ± 0.01 1/h and *μ_CB,max_* = 0.54 ± 0.04 1/h. Among the strains with slow cellobiose consumption, DSM No 2314 had an average uptake rate of *μ_CB,max_* = 0.034 ± 0.01 1/h while the strains DSM ID 14‐300 and DSM ID 14‐298 had higher uptake rates of *μ_CB,max_* = 0.07 ± 0.04 1/h and 0.07 ± 0.04 1/h, respectively.

With the rise in toxic power, the intensity of inhibition increases for a determined lactate concentration. With n > 1, the inhibition term shows a hyperbolic behaviour. This is evident from the slow process performance, low decrease in carbohydrate concentration and lactic acid formation towards the end of the cultivation.

Comparing the strains, the parameter of toxic power shows a lower impact of the lactate concentration towards strain DSM No 2314, with an average value of n = 1.37 ± 0.08. That suggests a slightly more linear dependence on lactic acid inhibition. The other strains had higher values, with an overall total average of n = 1.80 ± 0.13.

## 5 Discussion

During the last years, thermotolerant or respectively thermophilic (50–60 °C) l-(+)-lactic acid producing MOs gained much interest, such as *B. coagulans*. Several studies concerning different Bacillus strains, isolated from nature, were published. *B. licheniformis* TY7 [46], *B. licheniformis* BL1 [47], *B.* sp. 2–6 [48], *B.* sp. XZL9 [49], *B. coagulans* CCM 4318 [50], *B. coagulans* 36D1 and P4-102 B [51], *B. coagulans* SIM-7 [52], *B. coagulans* MXL-9 [53], *B. coagulans* WCP10-4 [45].

Effectivity in high lactic acid titers, yield, and productivity are essential for commercially cost efficient lactic acid production e.g. reducing downstream processing costs by high substrate concentration tolerance [45]. The *B. coagulans* strain, discussed in this study, gave a total average LA yield concentration of 87.7 ± 5.3 g/L among all examined strains in a simple batch fermentation process. This equals 67.4 ± 3.7% of LA yield for the total used amount of carbohydrates and 85.4 ± 4.7% of LA yield based on the amount of metabolized carbohydrates. This yield is at least in the same range or higher than the ethanol or lactic acid production described by other researchers.

Otto (2004) [54] described the strains of *B. coagulan*s DSM No. 2314, DSM No. 2319 and *B. smithii* DSM No. 459 and DSM No. 460 grown in lignocellulosic sugar containing medium. Otto (2004) [54] described a yield of 35 g/L (70 % LA) from 50 g/L xylose as the sole carbon source. Ou et al. (2011) [31] described a LA production by batch fermentation using the *B. coagulans* strain 36D1 up to a yield of 60 g/L with a significant residual glucose amount. Only a yield of 40 g/L LA was achieved using xylose as carbohydrate source [31]. Lactate formation by fermentation of glucose and growth of *B. coagulans* is known to be inhibited by high substrate concentrations – e.g. initial glucose concentration higher than 100 g/L may lead to prolonged lag time and decrease of growth rate [52] – and the product concentration as high lactate concentrations lead to unfermented carbohydrates at the process end. Higher production rates of LA in fed-batch processes are also typical suppressed by end-product inhibition therefore batch fermentation is still the most commonly used method for industrial LA production, although it has relatively low productivity due to end-product inhibition [52]. By using CaCO_3_ in the fermentation medium to overcome the lactic acid inhibition the yield could be increase concentration to 110 g/L from glucose and 120 g/L from xylose [31]. Here in this presented study, the used *B. coagulans* strains gave a yield about 90 g/L without any additional methods to overcome LA inhibition. However, fermentation processes using either high initial substrate concentrations, e.g. up to 240 g/L glucose or 200 g/L corn starch for simultaneous saccharification and fermentation processes (SSF) [45] are as important as the possibility for co‐fermentation of different substrates glucose, xylose, arabinose, and cellobiose.

Several LA producing bacteria, were reported to be able to utilize lignocellulose derived sugars. The strain *B. coagulans* MXL 9 was reported by Walton et al. (2010) [53], for its ability to utilize a hemicellulose water extract of mixed southern hardwoods. Wang et al. (2011) [47] described a fermentation process for l-(+)-lactic acid produced from 195 g/L xylose using *B. coagulans* XZL4 (DSM No. 23183), and *B. coagulans* XZL9 (DSM No. 23184). Van der Pol et al. (2016) [55] reported about *B. coagulans* DSM No. 2314 using 72.6 % glucose, 24.2 % xylose, 3.2 % galactose in 100 g/L Medium composition. Furthermore, LA producing *Lactobacillus delbrueckii* Mutant Uc-3 was described [56]. Xu et al., 2013 [57] used the *B. coagulans* strains XZL4 (DSM No. 23183) and XZL9 (DSM No. 23184) for l-(+)-lactic acid production fermenting pentose or hexose as carbon source. The highest amount of l-(+)-lactic acid was produced from glucose with 173 g/L, while 195 g/L were produced from xylose. The yield was up to 98 %. The production of l-(+)-lactic acid from reducing sugars in xylitol byproducts was presented with 106 g/L. Wu et al. (2014) [58] present information related to *B. coagulans* strains C106, JI12 and WCP10-4. In batch fermentations it was shown that 66 g/L LA was produced from xylose by *B. coagulans* JI12 with a yield, of 91%. *B. coagulans* C106 produced 101 g/L LA with a yield of 94 %. The strain WCP10-4 produced 70 g/L of LA from 75 g/L of xylose giving a yield of 96 %. The strain *B. coagulans* MXL 9 was used in the study described by Walton et al. (2010) [53], where its ability to fully utilize a hemicellulose water extract of mixed southern hardwoods led to an LA yield of 94 %. An LA yield of 81 % was specified by Maas et al. (2008) [59] for *B. coagulans* DSM No. 2314 fermenting a hydrolysate of lime-treated wheat straw.

There are a few reports available on LA producing bacteria which are able to use cellobiose for l-(+)-lactic acid production. While Abdel-Rahman et al. (2011) [60] described *Enterococcus mundtii* in more detail Adsul et al. (2007) [56] described *Lactobacillus delbrueckii*. Normaly these strains grow in a mesophilic temperature range of 30 to 43 °C. These temperatures do not represent optimal conditions in the thermophilic range of 50 to 55 °C for cellulases including β-glucosidases. Processes performed in the optimum range for cellulases performance and lactic acid by the used MOs should be more effective and economical efficient. However, only few data is available about the fermentation of cellobiose by *B coagulans*. Ong et al. (2016) [61] described the thermophilic *B. coagulans* WCP10-4 was found to be able to convert cellobiose. They reported that *B. coagulans* WCP10-4 converted 200 g/L of cellobiose to 196.3 g/L of l-lactic acid, equals a yield of 97.8%, without supplementation of external β‐glucosidases. This characteristic indicates that the *B. coagulans* WCP10-4 strain is an efficient strain for cellobiose conversion to l-(+)-lactic acid. Within this study it was possible to present two additional *B. coagulans* strains which are able to utilize cellobiose to a high extend.

The here presented *B. coagulans* strains have the benefit of high LA titers and productivity using glucose, xylose, and cellobiose for fermentation.

The strains with the DSM ID 14–301 and DSM ID 10–395 showed a high performance in their ability to consume an average of 9.8 ± 0.6 g/L xylose and to utilize cellobiose to a high extend with an uptake of 24.3 ± 2.5 g/L. However, the strains with the DSM ID 14‐300 and DSM ID 14‐298 are comparable to the lower performance of the DSM No. 2314. Combined, these three strains are able to metabolise a total average of 15.5 ± 1.9 g/L xylose and 6.3 ± 1.7 g/L cellobiose.

Thus, DSM ID 14‐301 and DSM ID 10‐395 were identified as capable of metabolizing glucose, xylose, and cellobiose throughout the process but showed a preference towards cellobiose consumption. Therefore, those strains are the most interesting for further studies characterizing their hydrolytic activity on a genetic basis.

Since cellobiose is a potent inhibitor the possibility to use strains that can directly utilize cellobiose for lactic acid production would be beneficent to lower the cellobiose inhibition on cellulases and reduce the enzyme cost e.g. in SSF.

Often, intermediate lag phases, indicating the change in metabolism for different sugar kinds, are described e.g. for *B. coagulans* MXL‐9 [53]. Within the used strains, intermediate lag phases were not detectable. This can be clearly seen in the online measurement of the alkaline addition.

The strains used in this study showed simultaneous consumption of glucose, xylose, and cellobiose, as these sugars were in a similar concentration range in the growth medium. Such a simultaneous consumption of carbohydrates at nearly equal concentrations was also shown in a fermentation process using 50 g/L glucose and 53 g/L xylose for the strain *B. coagulans* JI12 [62]. Additionally, data for a simultaneous metabolism of glucose and xylose at equal concentrations was described [63]. These results are also consistent with the study of Glaser and Venus (2017) [39] using glucose, xylose and arabinose in an artificial fermentation medium at different concentrations. Furthermore, a study discussed that *B. coagulans* DSM 2314 showed a simultaneous utilization of glucose and xylose during lactic acid production [59].

The most common technical use of LA is for the synthesis of poly-lactic acid (PLA). For the synthesis of PLA, only optically pure L- or d-lactic acid monomers can be used as precursors. Therefore, the production of optically pure l-lactic acid or d-lactic acid is a very important prerequisite for polymer synthesis.

The optical purity of l-(+)-lactic acid produced by the strains in this study was previously discussed by Glaser and Venus (2017) [39] and given for strain DSM No. 2314 at 98.9%, DSM ID 14‐298 at 98.9 %, and DSM ID 14‐301 at 99.6%, strain DSM ID 14‐300 had an optical purity of 99.9 % of l-(+)-lactic acid. The strain with the DSM ID 10‐395 could achieve a l-(+)-lactic acid of 95.9% in a test screening (unpublished data).

For comparison, Otto (2004) [54] described an optical purity of 96.7 to 99.7% of their l‐(+)‐lactic acid produced by strains of *B. coagulans* DSM No. 2314, DSM No. 2319, *B. smithii* DSM No. 459 and DSM No. 460. Xu et al. (2013) [57] proposed for their fermentation process of glucose, xylose and xylitol an optical purity is over 99 %, using *B. coagulans* strains XZL4 (DSM No. 23183) and XZL9 (DSM No. 23184).

Interesting and yet rarely discussed is a short yield comparisons of LA production with the production of other chemicals, such as ethanol, butanol, and acetone. For example, Ko et al. (2009) [64] described a yield of 83.1 % (12.7 g/L) of ethanol by *Saccharomyces cerevisiae D5A* using aqueous-ammonia pre-treated rice straw. Muñoz et al. (2015) [65] described an ethanol yield of 51 % (35 g/L) of glucose conversion using an ethanol/water-based organosolv-treated *Eucalyptus globulus* tension wood that previously resulted in up to 69–77 % saccharification yield. Amiri and Karimi (2011) [66] used an ethanol/water treatment of pine, oak, and elm wood that resulted in a yield of 73 % total sugar and 11.6 g/L of combined acetone, butanol, and ethanol yield. A 70 % yield of d-lactic acid by *Lactobacillus delbruecki* using hydrolysed starch powder was reported [64]. These studies indicate the large differences in productivity of different bio-based chemicals by other organisms. This gap in productivity and the possibility to utilize several carbohydrate sources show a further advancement of a production of the biochemical of lactic acid beneficent.

Lignocellulose as an abundant and renewable resource and its conversion has attracted much attention for the production of chemicals such as lactic acid. Still challenging is the cost-efficient pretreatment providing fermentable sugars. In this context, starchy materials are reported to remain the major carbon sources for the production of LA in future [67]. Therefore there is still a strong need for cost-effective processes for LA production from lignocellulose sugars e.g. by using newly isolated *Bacillus coagulans* strains with very high LA titre, productivity and yield or optimization of commercial feasible applications.

However, the tested *B. coagulans* strains provide a strong argument for efficient LA production using mixed carbohydrates from lignocellulosic sources. The results presented above show that *B. coagulans* has a rapid fermentation rate at 52 °C leading to production up to 90 g/L of L(+)-lactic acid in a normal batch process.

The fermentation processes of the different strains used in this study showed their capability to consume glucose, xylose, and cellobiose while enduring the presence of inhibitory compounds from an organosolv pre-treatment. Several strains also were resistant to higher lignin concentrations and have also been shown to be able to uptake lignin from the growth medium [39]. These strains showed the best properties for use in lignocellulose hydrolysates consisting of different carbohydrates. The production of l‐(+)-lactic acid by *B. coagulans* as a bio-based chemical provides the possibility to produce LA in high yields through the effective and efficient metabolization of available sugar components. This enables a more cost-effective production of LA than is currently available for other chemicals. The proposed model equations and parameter reduction made it easy to derive the basic kinetic key parameters for strain comparison without the need of a previous determination of additional parameters by costly screenings. The parameters derived by the fitting of the model to the experimental data showed a good possibility for interpretation along with the parameters derived by the fermentation process.

## 6 Conclusions

Using an organosolv hydrolysate fermentation process, five *Bacillus coagulans* strains were compared for their basic key performance parameters to produce lactic acid. A proposed kinetic model used to derive the basic key performance indices was able to reflect the growth behaviour very well despite the high degree of parameter reduction. The tested strains demonstrated good performance in fermenting the organosolv hydrolysate soluble sugars. Two strains displayed good performance in cellobiose utilization, but the xylose consumption was lower compared to other three strains exhibiting a higher xylose uptake rate but lower consumption of cellobiose. The results indicate that it is beneficial to co-cultivate strains that are good pentose consumers with strains that perform cellobiose uptake effectively for high yield production of lactic acid.
